# Pediatric cardiac care for the economically disadvantaged in India: Problems and prospects

**DOI:** 10.4103/0974-2069.52810

**Published:** 2009

**Authors:** Shyam S Kothari

**Affiliations:** Department of Cardiology, All India Institute of Medical Sciences, Ansari Nagar, New Delhi - 110 029, India

There is no fundamental difference in the care of children from economically disparate backgrounds. Yet, the questions of access, affordability and quality of care are real. Health is a fundamental right of all citizens. What does one do with this rhetoric when 95% of all infants requiring cardiac surgery are not currently getting it – and not all of it is because of lack of money. In such a scenario, we, the members of the Pediatric Cardiac Society of India, are forced to deliberate on issues that are somewhat “alien” to us, such as health care systems, resource allocation, ethics, prioritization, etc. It is beyond the scope of the article as well as the capacity of the author to suggest a right course of action. Nevertheless, a few reflections might help in providing a perspective on some of the questions raised by Maheshwari *et al*. in this issue of the Journal.[1]

## THE GROUND REALITIES

The lack of trained manpower, limited institutions and humongous “calculated demands” are well known.[2,3] Restricting only to the congenital heart disease (CHD) presently, the following demographics for India are relevant [Table 1].

**Table 1:** Demographics of India

Population	1.14 billions
Crude birth rate	22.2% in (2008)
New borns with disease t @ 8/1000 live births	∼ 2,00,000/yr
New borns with serious heart disease @ 2/1000 live births (most conservative estimate)	50,000/yr
Infant mortality	84 in 2000
	65 in 2000
	58 in 2007
GDP/capita (USD)	1508 in 2000
	3452 in 2008**
GDP rank	(161/225 nations)*
Human development	132/175 available nations*
Index ranking	
Population below poverty line	∼ 27%
No. of cardiac surgery for CHD estimated	6000 in 2001, about 10,000 now#
No. of infant cardiac surgeries estimated	1200 in 1998, 2500 in 2007 (ref 2)

* Data accessed at http//hdr.undp.org;

** http//www.indexmundi.com/India;

# estimated

While CHD remains the most common cause of neonatal mortality from congenital diseases, there are multiple prevalent primary health care issues that are responsible for more than 90% of all infant deaths. These sobering statistics are actually quite engaging to pediatric cardiac personnel. The impressive fall in infant mortality rates, rise in GDP and steady all round improvements augur well for children with CHD. Slowly but surely, CHD is gaining grounds as health care priority but yet has miles to go.

## SHOULD PEDIATRIC CARDIAC CARE BE CONSIDERED A PRIORITY?

This question has often been misunderstood and has been asked and answered in different domains. While it would be foolish to take away any emphasis from the importance of primary health care in public health, pitching primary versus tertiary is useful only in some limited sense. Simply put, the treatment of heart defects saves lives, and saves them cost effectively. The public health measures like immunization, sanitation and clean drinking water are essential in any case and save many more lives. The primary care versus tertiary care is not in a temporal sense, nor are they mutually exclusive. The concept of pediatric cardiac care is undeniably sound, the operational issues of how best to do it in our setting is a challenge. It would not be out of place to point out that nearly 2/3 of congenital cardiac defects could be considered “nearly cured,” with a life expectancy touching normal, or only mildly abnormal. The yard stick of cost-effective intervention in the USA is 50,000 US dollars for 1 year of quality-adjusted life year and, according to the NICE guidelines, it is about 20,000 pounds in England.[4,5] These societies have GDPs 10 times or more and health expenditure 100 times more than ours. The World Health Organization accepts a regional definition of cost effectiveness per quality-adjusted life year as any intervention costing about the GDP of the nation as very effective and within three times GDP as effective for that nation.[5] Thus, the treatment of CHD is easily an effective intervention conceptually by using public health yardsticks. However, individually still, more than y 50% of the families may not be able to afford the costs and if everybody could afford it, we do not have the manpower to meet more than 10% of the current demands. There are no magic bullet-type smart solutions to this problem. Capacity building of any nature is an arduous conscious effort.

## HEALTH CARE SYSTEMS

Every health care system has three components - resources, risk sharing and delivery mechanisms. Arguments about the best ways to handle each of the three aspects are a legion and continuous. It is instructive to analyze the situations in other parts of the world. In a World Bank publication,[6] an attempt to identify factors associated with good health care systems in low- and middle-income countries [Table 2] revealed interesting facts. There was no one model for best health care delivery. Instead, successes (and failures) were linked to the country's political, historical, cultural, geographical and societal variables. Political stability, economic growth, strong institutional and policy environment, focus on primary care, carefully sequenced health service delivery, commitment to equity and solidarity were some of the key elements in success. While these results are intuitive, they underscore the fact that progress in medicine is so much interlinked to progress in infrastructure, fiscal growth and other human development indices even though one does not guarantee the other. As Virchow famously remarked, “… medicine is a social science, and politics is nothing but medicine on a grand scale.”

**Table 2:** Good health system countries compared with India

Country	IMR	Doctors/1000	Life exp.	GDP/capita (USD)	Overall health spending % GDP	Govt. spending on health as % of total health spending
Sri Lanka	12	0.55	75	1033	4.3	45.6
Vietnam	17	0.53	71	550	5.5	27.1
Thailand	18	0.37	71	2539	3.5	64.7
Tunisia	21	1.34	73	2832	6.2	52.1
Costa Rica	11	1.32	79	4349	6.6 (Costa Rica)	77.0
Columbia	18	1.35	73	2155	7.8	86.0
Chile	8	1.09	78	5894	6.1	47
Estonia	6	4.48	73	8328	5.3 (Estonia)	76
Kyrgyz Rep.	58	2.51	68	434	5.6	40
India	58	0.6	64	3245	5	20

(adapted from ref 6)

It is also noticeable that pediatric cardiac care in most of these good health models is still worse than that in India. This is the result of the schism between primary and tertiary care approaches.

## RESOURCES

Let us think if as a society we have monetary resources for the treatment of CHD? In India, the government spends only 1% (increasing to 2% slowly) of the GDP on health. Overall, nearly 5% of the GDP is spent on health. The annual budget for the year 2008 allocated Rs. 165,340 millions to health, of which about 75% was slated for the rural health mission.[7] The integrated Child Development Scheme received Rs 63,000 millions; unfortunately, it did not include any allocation for the treatment of CHD. If we can have the fiscal space for a Rs 60,000 crore waiver for farmers' debt, we could certainly afford Rs 200 crore consumables required for CHD treatment of all newborns in a year (hypothetical calculation with expenditure of Rs 1 lakh/operation and 50% spending by the government). In the current environment, in policy terms, money by itself is not the only bottleneck for CHD. Of course, resources can be generated by other non-governmental sources. When it comes to children, we, and every nation, would do the extra effort required to garner the required monetary resources.

## RISK SHARING

The strength of any community's/nation's health care program stems from the solidarity of its citizens and willingness to share the wealth for the health of its members. “The test of our progress is not whether we add more to the abundance of those who have much; it is whether we provide enough to those who have too little,” said Franklin Roosevelt. In India, 80% of the costs of medical treatment are borne by out-of-pocket expenditure. In CHD management, the costs (medical and non-medical) are beyond the reach of most individuals and some mechanisms of risk sharing need to be put in place. Technically, how best to do risk sharing is debatable even as seen in the recent US elections. Clearly, insurance managed by private players is not the solution. Even the public-private partnership initiative experiences in Sweden and Great Britain have been disappointing.[8] Although country specific, these lessons should not be forgotten. Government involvement in health in a staged, transparent, committed manner appears appropriate. But, a strong institutional base is required. In the past, in the Indian setting, only 16% of the slated money is said to have reached the poor. Government money is liable to, although not destined to, abuse and corruption. Thus, a policy of all children operated at all hospitals at government expenditure may not to be sustainable. The government-initiated program of health insurance in 2004 had a lukewarm response. The current Rashtriya Swasthya Bima Yojana is primed to give Rs 30,000 to people below poverty line for any health-related expenditure that falls short for CHD costs. A great beginning seems to have been made in the governmental initiative on health in the Rural Health Mission that incorporates all the sane administrative principles focused on primary care. The country is watching expectantly and its success will help further progress. Risk sharing by many other non-governmental mechanisms, and social groups, may be convened in larger or smaller units.

## HEALTH CARE DELIVERY

The health care delivery mechanisms in CHD have to rely on hospitals and health care workers. Currently, the lack of available delivery systems is the biggest challenge. But, the access to the existing ones need to be streamlined and made simple. Further, the resourceless patients in high-demand tertiary care environments fare particularly poorly and need special protection beyond medical care. The care givers have to be sensitive to these aspects. The government-controlled health care mechanisms are prone to inefficiency, corruption, long waiting lists and license Raj and unregulated private “for profit” institutions can increase the delivery costs for the common man and often usurp the tax payer's money surreptitiously. Better media management and the freedom to practice “Boutique medicine” facilitate their role further. The final shape of the health care delivery system reflects the sum total of the society's wisdom. The stakeholders in the CHD are not large as the speciality is labor intensive and profit margins are modest in financial terms.

The shortage of manpower is real. The growth of manpower for hospitals is likely to show with time as the number of undergraduate doctors have increased from 11,000 in 1990 to 26,000 in 2008.[9,10] Equal increase in superspecialists, nurses, technicians and equal improvements in quality is not assured however.

The role of telemedicine in facilitating the access and treatment seems particularly suited to the Indian environment and should be utilized more systematically.

## RESOURCE ALLOCATION

Appropriate resource allocation within the population is a perennial problem because resources are never infinite. Doctors often like to argue on pure technical grounds and evade such debates, but we should remember that there are no value-free “pure” medical decisions. With respect to treatment of CHD in India, it may be worth browsing through some of the issues raised in the recent article in the Lancet on different principles of allocation of very scarce resources.[11]

The pros and cons of four different methods: (a) treating people equally, (b) favoring the worst off, (c) maximizing the total benefit and (d) promoting and rewarding social usefulness were presented.

As the authors point out, treating people equally is not really fair as it ignores many other relevant principles. Favoring the sickest is also not enough as it converts chronic to acute sickness. Again, no single moral or ethical principle can solve every dilemma. They suggest an alternative system – the complete lives system - which prioritizes younger people who have yet not lived a complete life and also incorporates prognosis to save most lives. By this token, pediatric cardiac services should get more priority than what they are currently getting. In this scheme, although infants got preference over old-aged people, children were preferred over neonates.

We should also use it to deliberate on the question of prioritizing within CHD. Is it ethical to suggest prioritizing the treatment of the “Curable” CHD over the complex cases that are less likely to result in satisfactory outcome for the individual, family and the society? Is it prudent to refrain from treating single ventricle with pulmonary atresia or heterotaxy and complex CHD from public funding? Should we tag the governmental subsidy to the families having only 1-2 children hence rewarding socially acceptable behavior? Should we reward the treatment of the girl child financially so that the dismal neglect of girls with CHD is partially offset? These are some examples of the questions that we all “stumble upon and quiver to answer daily.” Whichever system we follow, it is better to debate and have a pragmatic central guiding set of rules for a length of time that can be reviewed and revised rather than have no rules at all.

## WHAT SHOULD WE DO AS CLINICIANS?

We must streamline our systems and our thoughts to be able to deliver effective care to the less-resourceful patients, more than what is customary.[12] It is clichéd but important that we should “think globally but act locally.” We should be sensitive to local dynamics, local realities and should engage ourselves in research, innovations and advocacy accordingly. Opportunities for garnering local resources are lost in the absence of an active advocacy group. In terms of academic pediatric cardiac care, there are bound to be a multitude of factors waiting to be discovered. It is naïve to think that thalidomide episode was the only aberration. The management pathways for our patients need not be identical to those pursued elsewhere. Each center must seriously decide what is best for its patients.

Resourcelessness of our patients makes them stoic and trusting and that increases our responsibility several folds.

The selection of appropriate technology is also important. It is somewhat disheartening to note the speed with which we imbibed the costly device closure technology. Industrial support for indigenization is scant, but is bound to develop, and we should try to catalyze it. Inter-regional research is conspicuous by its absence for a country of this size. Continuous improvements in health care delivery require clinical audits, open minds and an attitude of striving toward excellence and perfection.

In the end, I wish to point out that the stresses and strains that we suffer in our professional career currently are enormous. A combination of huge workloads, a continuous lack of resources, especially in dealing with the economically disadvantageous population, and travails and triumphs of teamwork all combine to create waves of hope and despair for all of us.[13] But, do not forget that such opportunities as we currently have are unparalleled in the rest of the world. Not many professionals have their hands as full as ours – so “make hay while the sun shines,” albeit too brightly. “The character of a society is judged by the way it treats it's children and mute animals” said Mahatma Gandhi. Hopefully, our grandchildren will judge us fondly.
